# Molecular epidemiology and antibiotic resistance profiles of invasive *Haemophilus influenzae* from Norway 2017–2021

**DOI:** 10.3389/fmicb.2022.973257

**Published:** 2022-08-29

**Authors:** Ragnhild Tønnessen, Ignacio García, Nadia Debech, Jonas Christoffer Lindstrøm, Astrid Louise Wester, Dagfinn Skaare

**Affiliations:** ^1^Department of Infection Control and Vaccines, Norwegian Institute of Public Health, Oslo, Norway; ^2^European Public Health Microbiology Training Program (EUPHEM), European Centre for Disease Prevention and Control (ECDC), Stockholm, Sweden; ^3^Department of Bacteriology, Norwegian Institute of Public Health, Oslo, Norway; ^4^Department of Method Development and Analytics, Norwegian Institute of Public Health, Oslo, Norway; ^5^Department of Microbiology, Vestfold Hospital Trust, Tønsberg, Norway

**Keywords:** *Haemophilus influenzae* (Hi), invasive, epidemiology, vaccine, antibiotic resistance, whole-genome sequencing (WGS), Norway, surveillance

## Abstract

Invasive *Haemophilus influenzae* (Hi) disease has decreased in countries that included Hi type b (Hib) vaccination in their childhood immunization programs in the 1990s. Non-typeable (NT) and non-b strains are now the leading causes of invasive Hi disease in Europe, with most cases reported in young children and the elderly. Concerningly, no vaccines toward such strains are available and beta-lactam resistance is increasing. We describe the epidemiology of invasive Hi disease reported to the Norwegian Surveillance System for Communicable Diseases (MSIS) (2017–2021, *n* = 407). Whole-genome sequencing (WGS) was performed on 245 isolates. We investigated the molecular epidemiology (core genome phylogeny) and the presence of antibiotic resistance markers (including chromosomal mutations associated with beta-lactam or quinolone resistance). For isolates characterized with both WGS and phenotypic antibiotic susceptibility testing (AST) (*n* = 113) we assessed correlation between resistance markers and susceptibility categorization by calculation of sensitivity, specificity, and predictive values. Incidence rates of invasive Hi disease in Norway ranged from 0.7 to 2.3 per 100,000 inhabitants/year (mean 1.5 per 100,000) and declined during the COVID-19 pandemic. The bacterial population consisted of two major phylogenetic groups with subclustering by serotype and multi-locus sequence type (ST). NTHi accounted for 71.8% (176). The distribution of STs was in line with previous European reports. We identified 13 clusters, including four encapsulated and three previously described international NTHi clones with *bla*_TEM–1_ (ST103) or altered PBP3 (rPBP3) (ST14/IIA and ST367/IIA). Resistance markers were detected in 25.3% (62/245) of the isolates, with *bla*_TEM–1_ (31, 50.0%) and rPBP3 (28, 45.2%) being the most frequent. All isolates categorized as resistant to aminopenicillins, tetracycline or chloramphenicol possessed relevant resistance markers, and the absence of relevant substitutions in PBP3 and GyrA/ParC predicted susceptibility to cefotaxime, ceftriaxone, meropenem and quinolones. Among the 132 WGS-only isolates, one isolate had PBP3 substitutions associated with resistance to third-generation cephalosporins, and one isolate had GyrA/ParC alterations associated with quinolone resistance. The detection of international virulent and resistant NTHi clones underlines the need for a global molecular surveillance system. WGS is a useful supplement to AST and should be performed on all invasive isolates.

## Introduction

*Haemophilus influenzae* (Hi) is a Gram-negative bacterium that can cause respiratory tract infections and invasive disease in humans (Van Eldere et al., 2014; Langereis and De Jonge, 2015; Slack et al., 2021). Hi can be transmitted from infected or colonized individuals by droplets, and the incubation period ranges from 2 to 10 days. Colonization may also lead to opportunistic or recurrent infections, in particular in immunocompromised or otherwise vulnerable persons (Lindemann et al., 2022). Hi are divided into typeable (encapsulated) and non-typeable (NT) (non-encapsulated) strains. Encapsulated strains can be divided into serotypes (a-f). Among these, Hi serotype b (Hib) is the most virulent. In year 2000, it was estimated that Hib caused more than eight million cases of severe disease and 371,000 deaths globally in children younger than 5 years (Watt et al., 2009). Hib meningitis is still prevalent in unvaccinated children in low-income countries, especially in infants (Slack et al., 2021). However, the occurrence of invasive Hib disease has declined dramatically in countries that introduced the Hib conjugate vaccine in their national childhood immunization programs in the 1990s (Van Eldere et al., 2014).

The capsular protein Polyribosyl Ribitol Phosphate (PRP) is the most important virulence determinant of Hib and is the main antigen included in the vaccine (Slack et al., 2021). The first vaccine developed against Hib was a PRP polysaccharide vaccine. Poor immunogenicity in infants and lack of protection against nasopharyngeal carriage was associated with use of this vaccine. To improve this, a conjugated protein-polysaccharide vaccine in which PRP is linked to a carrier protein (for example tetanus toxoid) was developed. The reduced occurrence of Hib (Van Eldere et al., 2014) led to an increase in non-b serotypes and NTHi. None of the Hib vaccines protect against these, since PRP is serotype-specific and NTHi are acapsular and hence lacks PRP.

In 2018, 3,982 confirmed cases of invasive Hi disease were reported in the EU/EEA, with highest incidence rates in children below 12 months and in people aged 65 years or above (ECDC, 2020). Most of these infections are now caused by NTHi strains. In Europe, the incidence of invasive Hi disease is reported to be highest in the Scandinavian countries (ECDC, 2020). A specific vaccine protecting against NTHi invasive disease is not yet available, since NTHi are more antigenically diverse which makes vaccine development more challenging (Slack et al., 2021). Treatment of severe, prolonged, and recurrent Hi infections depends on effective antibiotics. Penicillins or cephalosporins are often the first choice for empirical treatment (Tristram et al., 2007). It is therefore a concern that beta-lactam resistance is increasing (Tristram et al., 2007), and the WHO has listed Hi as one of 12 priority pathogens for research and development of new antibiotics (WHO, 2017).

Reduced susceptibility or resistance to beta-lactams in Hi occurs primarily through two well-characterized mechanisms (Tristram et al., 2007): (1) transferable beta-lactamases, most frequently *bla*_TEM–1_ causing resistance to penicillins, or (2) amino acid substitutions in the transpeptidase domain of penicillin-binding protein 3 (PBP3), encoded by the *ftsI* gene, which may affect susceptibility to all beta-lactams. Isolates with PBP3-mediated resistance (hereafter denoted “rPBP3”) are increasing worldwide (Van Eldere et al., 2014). Based on amino acid substitution patterns in 4 positions (385, 389, 517, and 526), rPBP3 Hi are categorized as low-level (low-rPBP3) or high-level resistant (high-rPBP3) (Tristram et al., 2007). Low-rPBP3 isolates, often categorized as susceptible to ampicillin by phenotypic methods (such as MIC determination) are prevalent in most of the world, while isolates with high-rPBP3 genotypes (hallmarked by the S385T substitution) are typically resistant to cefotaxime and frequent in some geographic regions (Skaare et al., 2014a,b; Van Eldere et al., 2014; Nürnberg et al., 2021). Importantly, the prevalence of Hi categorized as resistant to ampicillin and cefotaxime is affected by different clinical breakpoints for interpretation of beta-lactam MICs recommended by the European Committee on Antimicrobial Susceptibility Testing (EUCAST) (used in the present study) and the Clinical and Laboratory Standards Institute (CLSI).

Immunization against Hib became a part of the Norwegian childhood vaccination program in 1992 (NIPH, 2021b). In 2020, the vaccination coverage in 2-year-olds in Norway was 97% (NIPH, 2021a). Hi meningitis has been notifiable to the Norwegian Surveillance System for Communicable Diseases (MSIS) at the Norwegian Institute of Public Health (NIPH) since 1975. From 1993 all invasive Hi infections became notifiable. During the last decade, less than 10 Hib cases have been reported per year in Norway. From 2010–2017, the proportion of NT or non-b Hi among notified invasive Hi isolates in Norway has ranged from 92.2 (71/77) in 2016 to 98.6% (70/71) in 2014, and prior to the COVID-19 pandemic there seemed to be an increasing trend in the occurrence of these infections, especially among the elderly (NIPH, 2018).

The prevalence of rPBP3 isolates resistant to ampicillin (MIC > 1 mg/L) among non-invasive Hi in Norway increased from 2.5% in 2004 to 9% in 2007 (Skaare et al., 2014a). Although low-rPBP3 was the predominating genotype, 6% were non-susceptible to cefotaxime and 20% to meropenem. A specific substitution pattern (low-rPBP3 type IIA) was present in 41% of the rPBP3 isolates and associated with multi-locus sequence types (ST) 367 and ST14. Emergence and increase of clonally related high-rPBP3 isolates from 2006 to 2013 has also been reported (Skaare et al., 2014b). The molecular epidemiology and the mechanisms of antibiotic resistance in invasive Hi in Norway have not been investigated in the past using WGS data.

The NIPH holds the national reference laboratory for Hi in Norway and receives isolates from patients with invasive Hi disease for characterization. We studied invasive Hi infections in Norway from a 5-year period (2017–2021) using epidemiological data from MSIS and data from the reference laboratory. The objectives were to: (1) describe the epidemiology of invasive Hi infections in Norway, (2) characterize the molecular epidemiology of whole-genome sequenced isolates, (3) determine the prevalence of phenotypic antibiotic resistance, (4) assess the correlation between phenotypic resistance and genetic resistance markers, including chromosomal mutations associated with resistance to beta-lactams and quinolones, and (5) identify invasive Hi clones.

## Materials and methods

### Study design, samples, and data collection

**FIGURE 1:**
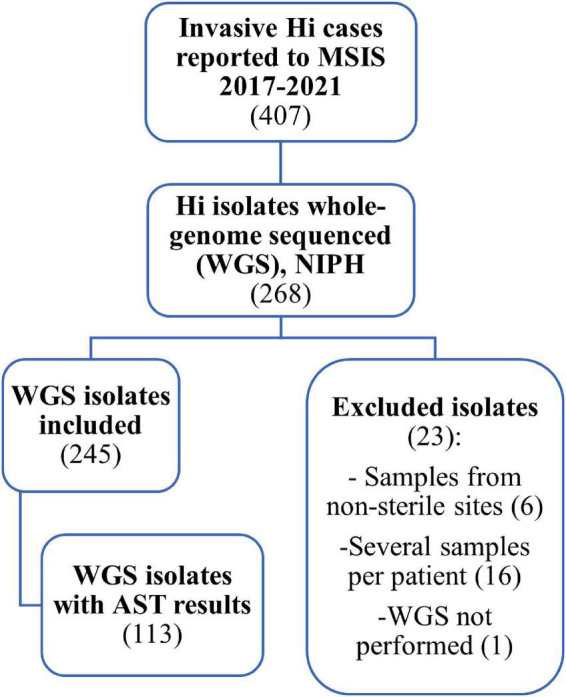
Flow chart showing the data used in the study of invasive *Haemophilus influenzae* in Norway 2017–2021. Of 407 cases reported to the Surveillance System for Communicable Disease in Norway (MSIS), we identified 268 samples submitted to the national reference laboratory at the Norwegian Institute of Public Health (NIPH) that were whole-genome sequenced (WGS). Of these, 245 isolates were included in the study. Data on antibiotic susceptibility testing (AST) was only available for 113 of the 245 isolates.

A retrospective descriptive study was performed. An overview of the number of cases and isolates included in the study is given in Figure 1.

To describe the epidemiology of invasive Hi infections in Norway, aggregated data on all 407 cases reported to MSIS 2017–2021 per sex, age, county, year and month was obtained (NIPH, 2022b). The case definition for reporting to MSIS is laboratory-confirmed Hi in normally sterile sample material by isolation or nucleic acid detection (NIPH, 2022a).

Primary laboratories are obliged to send samples (pure culture) from cases with invasive Hi to the reference laboratory at NIPH for confirmation and characterization (Lovdata, 2022). Whole-genome sequencing (WGS), using next generation sequencing (NGS) of Hi isolates was implemented at NIPH in 2017. On a routine basis, person-related and isolate-related data are registered in Laboratory information system (Labware), on arrival at NIPH.

Initially, a total of 268 whole-genome sequenced invasive Hi isolates were identified for the period 2017–2021 (Figure 1). We excluded 23 isolates: one from 2017 with lacking sequence, six collected from non-sterile sites (3 sputum, 2 throat, 1 nasopharynx), and 16 repeat isolates to limit the collection to one isolate per patient per disease episode. For patients with repeat isolates, we prioritized isolates originating from cerebrospinal fluid or fluids from body cavities over blood culture isolates.

**FIGURE 2:**
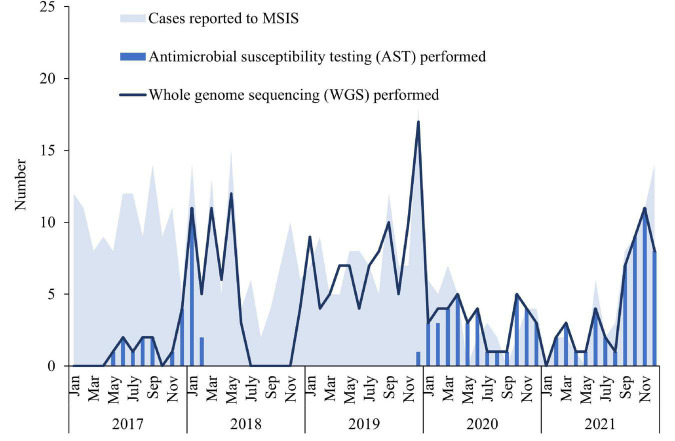
Number of cases of invasive *Haemophilus influenzae* disease reported to the Norwegian Surveillance System for Communicable diseases (MSIS) at the Norwegian Institute of Public Health (NIPH) by month in Norway 2017–2019 (blue shaded area, *N* = 407), compared to the number of isolates whole-genome sequenced at the national reference laboratory at NIPH included in this study (dark blue line, *N* = 245) and the number of isolates for which antimicrobial testing was performed (blue bars, *N* = 113).

A total of 245 isolates were included in the study. We obtained data from Labware on sex, age, and county of residence of the cases, as well as information about sample material, date of sample collection, and results from routine antimicrobial susceptibility testing (AST). AST results were available for only 113 of the 245 isolates since AST was not performed at NIPH from March 2018 to November 2019 due to lack of resources when NGS was implemented (Figure 2). For these 113 isolates, minimum inhibitory concentrations (MICs) for ampicillin, amoxicillin-clavulanic acid, cefuroxime, cefotaxime, ceftriaxone, meropenem, ciprofloxacin, tetracycline, chloramphenicol, and trimethoprim-sulfamethoxazole were determined using gradient test (Etest, bioMérieux). Isolates were categorized as Susceptible (S), Susceptible increased exposure (I), or Resistant (R), according to EUCAST clinical breakpoints (EUCAST, 2022). In addition, the isolates were screened for the presence of beta-lactamase resistance mechanisms according to EUCAST recommendations using the benzylpenicillin 1 unit (PG1) disk (until 2021 BD BBL Sensi-Disk, from 2021 Oxoid), with subsequent phenotypic testing of screening positive isolates for beta-lactamase production using nitrocefin (BD BBL Cefinase) (Livermore and Brown, 2001).

### Molecular characterization

For the 245 included isolates, WGS had previously been performed at the national reference laboratory at NIPH using the Illumina platform (MiSeq or NextSeq 550) and the KAPA HyperPlus library construction protocol. After removing the sequencing adapters using Trimmomatic (v 0.39) (Bolger et al., 2014), the bacterial genomes were assembled with SPAdes (v 3.15.2) (Nurk et al., 2013). Low quality contigs (i.e., k-mer coverage for the largest k < 2 and/or contig size < 500) were removed and Kraken2 (v 2.1.2) (Wood et al., 2019) was used to find and remove possible contaminant contigs. Hicap (v1.0.3) (Watts and Holt, 2019) was used to predict serotype, sequence type (ST) was predicted using sanger-pathogens/mlst_check (v 2.1.1706216), and *ftsI* allele numbers were assigned by pubMLST (Jolley et al., 2018). Genomes were annotated using Prokka (v 1.14.5) (Seemann, 2014) and core genomes were determined using Roary (v 3.13.0) (Page et al., 2015). Approximately-maximum-likelihood phylogenetic trees were built using FastTree (v 2.1.11) (Price et al., 2010). Microreact was used to analyze and visualize the data (Argimon et al., 2016).

MLST-*ftsI* typing with allele designations from pubMLST was used to define clones (Skaare et al., 2014a). In addition, assignment to core genome phylogeny-based clusters was done using branch lengths with an arbitrary, locally established cut-off for genetic distance.

The 245 genomes were screened for transferable antibiotic resistance genes using AMRFinderPlus and ResFinder in Abricate (v 1.0.0) with clean assembled contigs as input (Camacho et al., 2009; Zankari et al., 2017; Clausen et al., 2018; Bortolaia et al., 2020; Feldgarden et al., 2021). When phenotypic resistance was detected by AST, and no relevant transferable resistance gene was detected by Abricate, a search for the gene was performed using the annotations from Prokka.

Chromosomal alterations in proteins associated with increased resistance to beta-lactams (PBP3) or quinolones (quinolone resistance-determining regions (QRDR) of GyrA and ParC (aa 80–92) (Georgiou et al., 1996; Li et al., 2004) were identified by comparison with the reference sequence *H. influenzae* Rd KW20 (Fleischmann et al., 1995) through multiple sequence alignments of translated genes. Genotypic categorization of isolates with PBP3-mediated resistance (rPBP3) according to level (low- or high-rPBP3), stage (1–3) and group was done as described previously (Skaare et al., 2014b). In short, R517H or N526K were defined as first-stage substitutions (low-rPBP3, group I or II), S385T as second-stage substitution [high-rPBP3, group III-like(−) or III(−)], and L389F as third-stage substitution [high-rPBP3, group III-like(+) or III(+)].

To compare the 245 Norwegian invasive isolates with Hi worldwide, 802 available genomes in NCBI per 20.04.2022 were downloaded. Non-Hi sequences were identified using Kraken 2. After excluding the non-Hi samples, a total of 797 sequences were screened for presence of transferable antibiotic resistance genes. A phylogenetic tree was built using the same methods as described above.

### Statistical analysis

Incidence rates of invasive *H. influenzae* disease per 100,000 inhabitants in Norway overall, per age group, sex, county and year were calculated based on data from MSIS and population data from Statistics Norway (per 1 January each year) (SSB, 2022). Mean incidence rates for the entire period were also calculated. The number of Hi isolates per serotype, sex, age, and county was described. Maps were created with QGIS version 3.16.5.

The numbers and proportions of antibiotic resistance markers (genes and chromosomal alterations) and phenotypic resistance to the tested antibiotics (prevalence) were calculated. For the 113 isolates where both results from WGS and AST were available, we assessed the correlation between the presence of genetic resistance markers (transferable genes or chromosomal mutations) and phenotypic resistance by calculating sensitivity, specificity, positive predictive value (PPV), and negative predictive value (NPV) for each resistance marker. Isolates categorized as S or I were lumped as “susceptible” in the calculations.

## Results

### Descriptive epidemiology

A total number of 407 cases of invasive Hi disease was reported in Norway during 2017–2021. The overall mean incidence rate was 1.5 per 100,000 inhabitants/year and annual rates ranged from 0.7 to 2.3 (Supplementary Table 1). The number of reported cases to MSIS varied by month and year (Figure 2). There was a drop in the number of cases between February 2020 and July 2021, followed by an increase from August 2021. The mean incidence rate was highest in the oldest age groups; ≥ 90 years (12.4 per 100,000) and 80–89 years (7.9 per 100,000), but also relatively high among infants (< 1 year) (3.2 per 100,000) (Supplementary Table 1). The incidence rate increased with age from 60 years and older (Supplementary Figure 1). The mean overall incidence rate in females (1.7 per 100,000) was slightly higher than the rate in males (1.4 per 100,000), with an overall male/female ratio of 0.8 (Supplementary Table 2). The cases were reported from all 11 counties in Norway and the incidence rate varied by county and year (Supplementary Figure 2 and Supplementary Table 3). The highest mean incidence rates were found in Innlandet (2.7 per 100,000) and Agder (2.1 per 100,000) counties, whereas the lowest rates were found in Møre og Romsdal and Troms og Finnmark counties (both 1.1 per 100,000).

### Whole-genome phylogeny and molecular serotyping

Among the 407 cases reported to MSIS during 2017–2021, 245 whole-genome sequenced isolates were included in this study (Figure 1). The number of whole-genome sequenced isolates corresponded well with the number of reported cases to MSIS throughout most of the study period, except for some time periods where fewer isolates were sequenced (January to October 2017 and July to November 2018) (Figure 2). The isolates were obtained from blood culture (233), cerebrospinal fluid (9), pericardial fluid (1), peritoneal dialysis fluid (1) or pleural fluid (1). The median age of the cases was 64 years (mean 57 years, range 0–98 years) of which 58.8% (144) were female (Supplementary Table 4). The isolates originated from all 11 counties of Norway (Supplementary Table 5).

**FIGURE 3:**
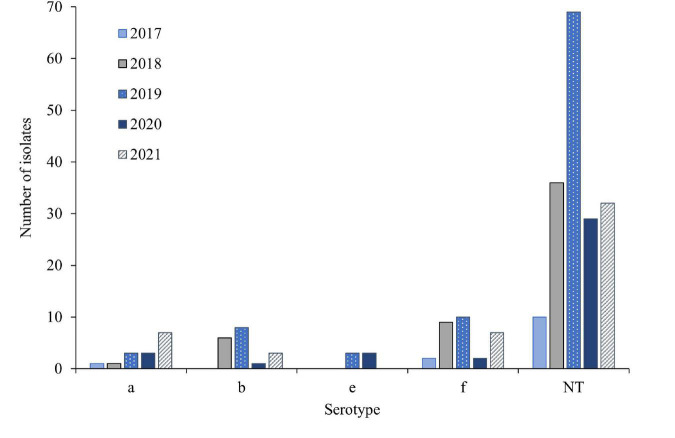
Number of Hi serotypes per year in 245 invasive *Haemophilus influenzae* isolates from Norway 2017–2021.

Overall, 71.8% (176) of the isolates were non-typeable (NT) (Figure 3 and Supplementary Table 6). Among the encapsulated isolates (69), 43.5% (30) were serotype f (Hif), 26.1% (18) were serotype b (Hib), 21.7% (15) were serotype a (Hia), whereas 8.7% (6) were serotype e (Hie). Serotypes c and d were not detected. The number of NT isolates dropped during 2020 and 2021. For Hif and Hib the numbers decreased in 2020 but increased again in 2021. In contrast, the number of Hia isolates increased from 2018–2021, whereas the number of Hie remained stable.

By MLST, the isolates belonged to 90 different STs (Supplementary File 1), with ST124 being most common (10.6%), followed by ST103 (8.2%), ST6 (4.9%), ST210 (4.5%) and ST23 (4.5%). No novel MLST alleles or profiles were detected.

**FIGURE 4:**
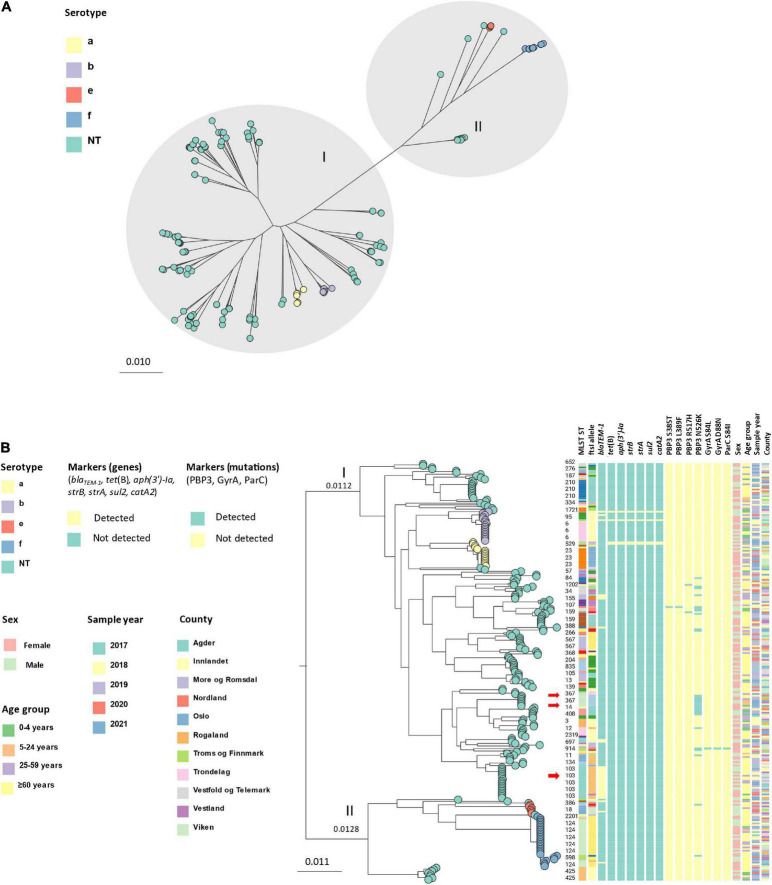
**(A)** Unrooted approximately-maximum-likelihood phylogenetic tree based on the core genome (core genes: 1158) of 245 invasive *Haemophilus influenzae* isolates from Norway 2017–2021. The isolates cluster in two main groups (division I and II, gray shading) and by serotype (leaf node color). **(B)** Midpoint-rooted approximately-maximum-likelihood phylogenetic tree based on core genome (core genes: 1158) annotated with division, serotype (leaf node color), ST, *ftsI* allele, markers of antibiotic resistance (genes and mutations), sex, age, sample year and county. The red arrows show clusters containing beta-lactam resistance markers (clusters 2, 8, and 11; Table 1).

Core genome phylogeny (core genes: 1,158, total genes: 5,942) showed that the isolates clustered in two major groups (Figure 4A), with a genetic distance of 0.0240 nucleotide substitutions per site between internal nodes (Figure 4B). The groups were identified as division I and II as defined by Meats et al. (2003) by comparison of serotype and ST distribution. The largest group (division I) contained 197 isolates, of which 164 were NTHi, 15 were Hia and 18 were Hib, whereas the other group (division II) contained 48 isolates; 12 NTHi, 6 Hie and 30 Hif. Within the two divisions, isolates clustered by serotype and ST. The genetic variation was generally larger among NTHi than among the encapsulated isolates. Overall, 73.3% (11) of the Hia isolates belonged to ST23, 66.7% (12) of Hib to ST6, 83.3% (5) of Hie to ST18 and 86.7% (26) of Hif to ST124.

**TABLE 1:** Cluster analysis based on MLST-*ftsI* typing and assessment of genetic distance by core genome phylogeny (Figure 4B).

Cluster^a^	Serotype	MLST profile^b^	*ftsI* allele^b^	Members (n)		
				MLST-*ftsI* typing^b^	Core genome phylogeny^c^	Both methods
1	Hif	ST124	*ftsI*-6	25	24	24
2	NTHi	ST103	*ftsI-*27	18	19	18
3	Hib	ST6	*ftsI*-10	12	12	12
4	NTHi	ST210	*ftsI*-37	11	11	11
5	Hia	ST23	*ftsI*-46	11	9	9
6	NTHi	ST425	*ftsI*-39	8	1	1
7	NTHi	ST159	*ftsI*-37	7	7	7
8	NTHi	ST367	*ftsI*-2	7	7	7
9	NTHi	ST567	*ftsI*-18	7	7	7
10	NTHi	ST12	*ftsI*-10	4	5	4
11	NTHi	ST14	*ftsI*-1	4	5	4
12	Hie	ST18	*ftsI*-55	4	5	4
13	NTHi	ST835	*ftsI*-8	4	5	4

^a^Only clusters with at least five members with at least one method are shown.

^b^Unique combinations of MLST profile and ftsI allele (MLST-ftsI typing) were used to define clones (Skaare et al., 2014a; Jolley et al., 2018).

^c^Cut-off for cluster definition: 0.0010 nucleotide substitutions per site (Figure 4B). By definition, clusters may comprise isolates with diverging MLST-ftsI types.

Cluster analysis by MLST-*ftsI* typing and core genome phylogeny-based clusters (using a cut-off of 0.0010 nucleotide substitutions per site) revealed 13 clusters with at least five members (by at least one method), accounting for 50% (123) of the isolates (Table 1). Four clusters comprised encapsulated isolates (Hia, Hib, Hie, Hif), with the Hif and Hib clusters representing the largest and third largest with 25 (10.2% of all isolates) and 12 (4.9%) members, respectively. In general, there was excellent correlation between the two methods for assessment of clonality. The only exception was cluster 6, consisting of eight isolates with identical MLST-*ftsI* type and genetic distances between the members of at least 0.0011 substitutions per site (i.e., exceeding the arbitrary cut-off for cluster definition). All clusters were heterogeneous with respect to age, county, and sample year, except clusters 7 (ST159) and 13 (ST835), which were exclusively found in elderly patients (≥ 60 years) (Figure 4B).

**FIGURE 5:**
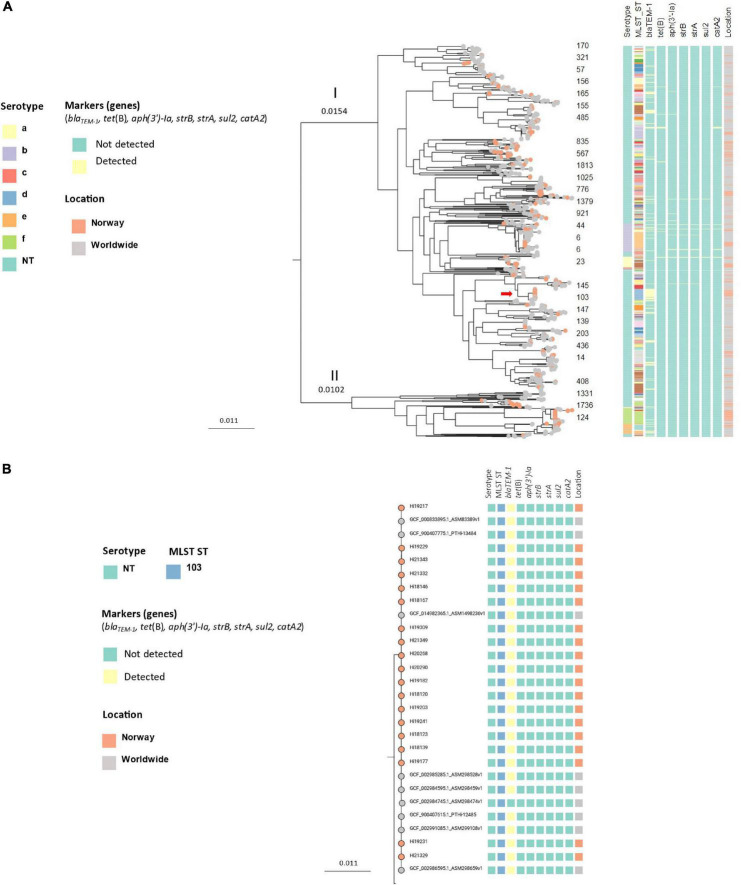
**(A)** Midpoint-rooted approximately-maximum-likelihood phylogenetic tree based on core genomes (core genes: 840) of 245 invasive *Haemophilus influenzae* isolates from Norway 2017–2021 (colored leaf nodes) and 797 publicly available sequences worldwide, annotated with serotypes, STs and transferable resistance genes. The red arrow shows the *bla*_TEM–1_-carrying ST103 NTHi clone. **(B)** Detailed presentation of the *bla*_TEM–1_-carrying ST103 NTHi clone [red arrow in panel **(A)**].

Core genome phylogeny of the Norwegian isolates and 797 worldwide Hi (core genes: 840, total genes: 11303) revealed a similar population structure, with a genetic distance of 0.0256 nucleotide substitutions per site between divisions I and II, and subclustering by serotype and ST (Figure 5A). Several of the Norwegian clusters formed distinct clusters in the worldwide tree, including the encapsulated clusters 1 (ST124 Hif), 3 (ST6 Hib), 5 (ST23 Hia), and 12 (ST18 Hie).

The phylogenetic trees with metadata can be accessed at Microreact: Invasive Haemophilus influenzae Norway 2017--2021^1^ and Invasive Haemophilus influenzae Norway 2017--2021^2^ compared to sequences worldwide.

### Antibiotic resistance markers

Overall, one or more genetic resistance markers were detected in 25.3% (62/245) of the isolates. Among these, 51.6% (32) had one or more transferable resistance genes in the absence of chromosomal resistance, and 45.2% (28) had altered PBP3 (rPBP3) in the absence of transferable genes. One rPBP3 isolate (1.6%) had a transferable resistance gene (*bla*_TEM–1_), and one rPBP3 isolate (1.6%) had additional alterations in QRDR of GyrA/ParC.

#### Transferable resistance genes

Seven different transferable resistance genes were identified among the 245 isolates (Table 2). Overall, 55 genes were detected in 13.5% (33/245) of the isolates. In two cases, AMRFinderPlus and ResFinder failed to detect a resistance gene (one *bla*_TEM–1_ and one *catA2*). We identified four different profiles (1–4), where profile 1 had no resistance genes, profile 2 had *bla*_TEM–1_ only, and profiles 3 and 4 contained six genes [*tet*(B), *sul2*, *catA2*, *aph(3’)-Ia*, *strA*, and *strB*] with or without *bla*_TEM–1_, respectively. The most frequent gene, *bla*_TEM–1_, was present in 12.6% (31/245) of the isolates, of which 83.9% (26) were NT, and 16.1% (5) were encapsulated (3 Hib, 1 Hie and 1 Hif).

**TABLE 2:** Antibiotic resistance gene profiles in 245 invasive *Haemophilus influenzae* isolates in Norway, 2017–2021, detected by ResFinder and AMRFinderPlus in Abricate.

		Transferable resistance genes (accession)^a^						
		*bla*_TEM–1_	*catA2*	*tet*(B)	*sul2*	*aph(3’)-Ia*	s*trA*	*strB*
Profile	N	(NG_050145.1)	(NG_047594.1)	(NG_048161.1)	(HQ840942)	(NG_047431.1)	(NG_056002.2)	(NG_047464.1)
1	212	–	–	–	–	–	–	–
2	29	+	–	–	–	–	–	–
3	2	–	+	+	+^b^	+	+	+
4	2	+	+	+	+	+	+	+
Total	245	12.7% (31/245)^c^	1.60% (4/245)^c^	1.60% (4/245)	1.60% (4/245)	1.60% (4/245)	1.60% (4/245)	1.60% (4/245)

^a^Coverage/identity 100/100% unless otherwise noted.

^b^Includes two strains with identity 99.88%.

^c^One bla_TEM–1_ and one catA2 gene not detected by Abricate, identified by sequence analyses.

Four isolates (profiles 3 and 4) carried genes associated with resistance to the non-beta-lactams chloramphenicol (*catA2*), tetracycline [*tet*(B)], trimethoprim-sulfamethoxazole (*sul2*), kanamycin [*aph(3’)-Ia*], and streptomycin (*strA* and *strB*) (Table 2). These were all encapsulated; two Hib (ST118 and ST1721) and Hia (ST4).

A clone of 19 ST103 NTHi isolates carrying *bla*_TEM–1_ was identified in division I by phylogenetic analysis (Figure 4B). The isolates were from 2018 (5), 2019 (8), 2020 (2) and 2021 (4) and submitted from 9 of 11 counties (no isolates from two counties in Northern Norway). When comparing the Norwegian invasive isolates with Hi worldwide, the Norwegian isolates clustered with non-Norwegian isolates (Figures 5A,B).

#### Substitutions in PBP3, GyrA and ParC

PBP3 substitutions used for rPBP3 genotyping were identified in 12.2% (30/245) of the isolates (Table 3 and Figure 4B). Based on substitution patterns, 29 isolates were classified as low-rPBP3 (2 group I and 27 group II), and one as high-rPBP3 group III(+). Overall, 24.5% (60/245) of the isolates had at least one molecular resistance mechanism (*bla*_TEM–_1 or rPBP3) against beta-lactams. One of the isolates had both *bla*_TEM–1_ and was categorized as low-rPBP3 group II. One of 245 isolates (0.4%) had substitutions within QRDR (aa 80-92) of GyrA (S84L and D88) and ParC (S84I). The same isolate (ST1859) was categorized as low-rPBP3 group II.

**TABLE 3:** Categorization of 245 invasive *Haemophilus influenzae* isolates (Norway 2017–2021) into rPBP3 genotypes based on amino acid substitutions in positions 385, 389, 517, and 526 compared to reference strain Rd KW20 (Fleischmann et al., 1995).

Level	sPBP3	Low	Low	High-	High-	High +	High +
Stage	0	1	1	2	2	3	3
Group	–	I	II	III-like(−)	III(−)	III-like(+)	III(+)
S385T	–	–	–	+	+	+	+
L389F	–	–	–	–	–	+	+
R517H	–	+	–	+	–	+	–
N526K	–	–	+	–	+	–	+
Number	215	2	27	0	0	0	1
%	87.8	0.8	11.0	0	0	0	0.4

We identified two rPBP3 NTHi clones with unique MLST-*ftsI* profiles and more than three members in division I (Figure 4B). One was ST14 (4), and the other ST367 (7) (Figure 4B). The clones shared identical PBP3 substitution patterns (D350N, M377I, A502V, N526K, V547I, and N569S). All ST14 isolates had *ftsI* allele 1, and all ST367 isolates had *ftsI* allele 2. These two clones together accounted for 36.7% of the rPBP3 isolates in this study.

### Phenotypic antibiotic resistance

AST results were available for 46.1% (113/245) of the isolates (Figure 1), sampled in 2017 (13), 2018 (13), 2019 (1), 2020 (37) and 2021 (49). Screening for beta-lactam resistance mechanisms (PG1 disk) was positive in 32.7% and beta-lactamase production was detected in 13.3% (15) of the isolates. Based on MIC values and clinical breakpoints, the 113 tested isolates were categorized as resistant to trimethoprim-sulfamethoxazole (21.2%), ampicillin (18.6%), cefuroxime (9.7%), tetracycline (2.7%), amoxicillin-clavulanic acid (1.8%), chloramphenicol (1.8%) and meropenem (0.1%) (Table 4). No isolates were categorized as resistant to cefotaxime, ceftriaxone, or meropenem.

**TABLE 4:** Summary of antimicrobial susceptibility testing (AST) (gradient) of invasive *Haemophilus influenzae* isolates from Norway 2017–2021 (*N* = 113).

Drug	MIC breakpoints (mg/L)^a^		Categorization of isolates% (n)			MIC_50_	MIC_90_
	S	R	S	I	R		
Ampicillin	≤ 1	> 1	81.4 (92)	–	18.6 (21)	0.5	7.2
Amoxicillin-clavulanic acid (iv)	≤ 2	> 2	98.2 (111)	–	1.8 (2)	0.5	1
Amoxicillin-clavulanic acid (po)	≤ 0.001	> 2	0.0 (0)	–	1.8 (2)	0.5	1
Cefuroxime (iv)	≤ 1	> 2	72.6 (82)	17.7 (20)	9.7 (11)	1	2
Cefotaxime	≤ 0.125	> 0.125	100.0 (113)	–	0.0 (0)	0.016	0.032
Ceftriaxone^b^	≤ 0.125	> 0.125	100.0 (112)	–	0.0 (0)	0.016	0.016
Meropenem (meningitis)	≤ 0.25	> 0.25	100.0 (112)	–	0.0 (0)	0.064	0.125
Meropenem (non-meningitis)^c^	≤ 2	> 2	100.0 (112)	–	0.0 (0)	0.064	0.125
Ciprofloxacin	≤ 0.06	> 0.06	100.0 (113)	–	0.0 (0)	0.008	0.016
Chloramphenicol	≤ 2	> 2	97.3 (110)	–	1.8 (2)	1	1
Tetracycline	≤ 2	> 2	97.3 (110)	–	2.7 (3)	0.5	1
Trimethoprim-sulfamethoxazole^d^	≤ 0.5	> 1	76.1 (86)	2.7 (3)	21.2 (24)	0.032	32
Beta-lactamase	Negative	Positive	86.7 (98)	–	13.3 (15)	–	–
Benzylpenicillin 1 unit^d^	≥ 12 mm	< 12 mm	67.3 (76)	–	32.7 (37)	14	20

^a^EUCAST clinical breakpoints version 12.0 (EUCAST, 2022).

^b^One sample excluded (likely error in MIC value, not retested).

^c^One sample missing/not tested for meropenem.

^d^Trimethoprim-sulfamethoxazole in the ratio 1:19. Breakpoints are expressed as the trimethoprim concentration.

^d^Screening for beta-lactam resistance mechanisms (disk diffusion).

### Correlation between antibiotic resistance markers and phenotypic resistance

The correlation between the presence of antibiotic resistance markers (acquired genes or alterations in chromosomal genes) and phenotypic resistance (MIC above the R breakpoint), expressed as test properties (sensitivity and specificity) and predictive values, is presented in Table 5. For ampicillin, calculations were performed separately for the two resistance markers (*bla*_TEM–1_ and rPBP3). In general, detection of resistance markers predicted phenotypic resistance with high sensitivities and specificities, and resistance to ampicillin and cefuroxime could in all cases except one (cefuroxime) be explained by *bla*_TEM–1_ and/or altered PBP3. However, detection of rPBP3 in *bla*_TEM–1_ negative isolates (98) had low PPV for ampicillin resistance (54.4%). The absence of a high-rPBP3 genotype predicted susceptibility to cefotaxime, ceftriaxone and meropenem with a NPV of 100% (not shown in Table 5 because no resistant isolates were detected). Detection of *catA2* and *tet(B)* explained all the observed phenotypic resistance toward chloramphenicol and tetracycline, and no susceptible isolates carried these genes.

**TABLE 5:** Prevalence of phenotypic resistance, and correlation between phenotypic antibiotic resistance and presence of genetic resistance markers (genes and mutations) in 113 invasive Hi isolates from 2017, 2020 and 2021, Norway, where both WGS data and AST data were available.

Group	Drug (n isolates)	Prevalence% (n)	Resistance marker	Resistance marker% (n)	Sensitivity%	Specificity%	PPV^a%^	NPV^b%^
Beta-lactams^c^	Ampicillin (all, *n* = 113)	18.6 (21)	*bla*_TEM–1_ and/or rPBP3^c^	23.0 (26)	100	94.6	80.8	100
	Ampicillin (rPBP3 neg., *n* = 101)	13.9 (14)	*bla*_TEM–1_	13.9 (14)	100	100	100	100
	Ampicillin (*bla*_TEM–1_ neg., *n* = 98)	6.1 (6)	rPBP3^c^	11.2 (11)	100	94.6	54.5	100
	Cefuroxime^d^, (*n* = 113)	9.7 (11)	rPBP3^c^	10.6 (12)	90.9	98.0	83.3	99.0
Tetracyclines	Tetracycline (*n* = 113)	2.7 (3)	*tet*(B)	2.7 (3)	100	100	100	100
Phenicols	Chloramphenicol (*n* = 113)	2.7 (3)	*catA2*	2.7 (3)	100	100	100	100

^a^Positive predictive value.

^b^Negative predictive value.

^c^Defined by the presence of N526K or R517H.

^d^Susceptible (S) and Susceptible, increased exposure (I) were lumped as “susceptible” in the calculations.

## Discussion

NTHi predominated as cause of invasive Hi disease in Norway, with the highest incidence rates in the elderly, followed by infants. This pattern has also been reported from other countries (ECDC, 2020). The overall incidence rate in Norway was comparable to the rates found in other countries (Soeters et al., 2018), but the incidence rate in infants was lower in our study. The reason for this is unknown but may reflect differences in population structure or testing practices. The population in Norway is aging (Brunborg, 2012), and the variation in incidence rates between Norwegian counties may also be explained by the different age distributions. The elderly are more prone to infections due to changes in the immune system associated with aging (immunosenescence) (Aw et al., 2007).

Core genome phylogeny is considered the gold standard for assessing genetic relationship between isolates and gives a higher resolution compared to conventional MLST based on seven housekeeping genes (Besser et al., 2018). Our phylogenetic analysis based on core genome showed that the population of Norwegian invasive Hi isolates consisted of two major phylogenetic groups, corresponding to division I and II, with subclustering by serotype and ST as described previously (Musser et al., 1986; Meats et al., 2003; De Chiara et al., 2014). NTHi was present in both divisions. We used two different approaches to assess clonality; MLST-*ftsI* typing (Skaare et al., 2014a; Jolley et al., 2018) and core genome phylogeny-based genetic distance with an arbitrary cut-off. In the absence of a standardized core genome MLST typing scheme for Hi, MLST-*ftsI* typing gives an unambiguous type assignment with higher resolution than MLST alone, allowing comparison of molecular epidemiology and distribution of clones across studies. There was excellent correlation between the methods, with one exception (ST425/*ftsI*-39) illustrating that MLST-*ftsI* types may be stable over time despite increasing genome-wide genetic distance caused by recombination and/or spontaneous mutations (drift), which may differ between phylogenetic lineages (Meats et al., 2003). This observation indicates that MLST-*ftsI* typing as the only approach has lower resolution and is inferior to core genome phylogeny for detection of local outbreaks. We have not been able to identify other published epidemiologic studies of invasive Hi utilizing the MLST-*ftsI* approach, but MLST-*ftsI* profiles for a large collection of international invasive and non-invasive Hi genomes (with metadata) are available in the pubMLST database^3^.

In our study, the population structure was polyclonal, with approximately 50% of isolates belonging to 13 clusters with at least five members. Coupling of molecular epidemiology with available epidemiologic data (sample year and county) did not reveal local clonal outbreaks, suggesting that the observed clusters represent endemic clones with the ability to persist and spread. Importantly, four of 13 invasive clusters belonged to STs (ST12, ST14, ST159, and ST367) ranging among the eight most frequent STs in a study of non-invasive (mainly respiratory) Hi in Norway in 2007 (Skaare et al., 2014a). This illustrates the importance of infection control measures for patients with respiratory tract infections to contain Hi, especially in nursing homes and other health care institutions hosting vulnerable patients (Andersson et al., 2015). The efficacy of such measures to reduce spread of Hi and lower the overall incidence rates of invasive Hi disease was demonstrated during the COVID-19 pandemic. Invasive Hi disease in Norway dropped from February 2020 to July 2021, corresponding to the period when several infection control measures toward COVID-19 were implemented (Seppälä et al., 2020). A similar reduction in invasive Hi disease during the pandemic was reported in several other countries (Brueggemann et al., 2021).

The STs of the major invasive Norwegian Hi clusters, except clusters 4 (ST210) and 13 (ST835), also predominate among invasive Hi isolates in Italy, Portugal, and France (Giufrè et al., 2018; Deghmane et al., 2019; Heliodoro et al., 2020). Furthermore, comparison with the pubMLST database showed that most Norwegian clusters had common MLST-*ftsI* profiles. Curiously, the Norwegian clusters were recognizable by worldwide core genome phylogeny but consisted in most cases strictly of Norwegian isolates. A possible interpretation may be that the Norwegian clusters represent regional variants of widely disseminated international clones with increased virulence. Detailed characterization of such clones is hugely important for vaccine development to reduce the burden of invasive Hi infections in the future. Hopefully, this work will be facilitated by identification of clones of interest through international collaboration projects, such as the recently established Invasive Respiratory Infections Surveillance (IRIS) Initiative, which allows comparison of molecular and epidemiological data for cases of invasive Hi across the globe^4^ (Brueggemann et al., 2021).

We observed an increase in the occurrence of invasive Hia, unaffected by the COVID-19 pandemic. A similar increase in invasive Hia has been reported from North America and some European countries (Topaz et al., 2022). It should be noted that the number of Hia in our study was small and it cannot be excluded that the observed increase was due to coincidence. Most of the Hia isolates in the present study belonged to cluster 5 (ST23/*ftsI*-46). This was one of four major Norwegian clusters of encapsulated isolates, of which the Hif clone (ST124/*ftsI*-6) alone accounted for 10% of the sequenced isolates. This confirms the previously reported high pathogenic potential of Hif strains (Resman et al., 2011), warranting further investigations to explore the molecular and biological mechanisms for the increased virulence of this clone.

Remarkably, we detected four encapsulated, multidrug-resistant (MDR) isolates carrying six or seven transferable resistance genes. Two out of 18 Hib isolates (10.5%) carried seven resistance genes associated with resistance to ampicillin, tetracycline, chloramphenicol, trimethoprim-sulfamethoxazole, streptomycin, and kanamycin. A similar combination of non-beta-lactam resistance genes was present in the genomes of two out of 15 Hia isolates (13.3%). These MDR profiles, to the best of our knowledge previously unreported in *H. influenzae*, suggest the presence of integrative conjugative elements (ICE) acquired through horizontal transfer (Hegstad et al., 2020; Johannessen et al., 2022). The molecular environment of the resistance genes and the presence of mobile genetic elements will be further investigated in a planned future study.

Resistance or decreased susceptibility to beta-lactams can be caused by beta-lactamase production or alterations in PBP3 (Tristram et al., 2007). As expected, *bla*_TEM–1_ was the most frequently detected resistance gene in the present study, with the *bla*_TEM–1_-carrying ST103 NTHi clone (cluster 2) as a significant contributor. The clone appears to be endemic in Norway and clustered with invasive Hi from Italy and Portugal (Giufrè et al., 2013; Pinto et al., 2019) by worldwide core genome phylogeny, consistent with an international, ampicillin-resistant NTHi clone associated with invasive disease.

Furthermore, an rPBP3 genotype was present in 12.2% of the isolates in the present study, compared to 15% in non-invasive isolates in Norway in 2007 (Skaare et al., 2014a). Low-rPBP3 group II was the most common genotype in invasive isolates, similar to in non-invasive isolates (Skaare et al., 2014a). Two out of 13 invasive clusters consisted of low-rPBP3 isolates with MLST-*ftsI* profiles similar to several genomes in the pubMLST database (cluster 8, ST367/*ftsI*-2; cluster 11, ST14/*ftsI*-1). Both clusters had a characteristic PBP3 substitution pattern previously assigned type IIA (Skaare et al., 2014a). Interestingly, ST14/IIA and ST367/IIA were the predominating rPBP3 clones among non-invasive Norwegian Hi in 2004 and 2007, respectively, and the two clones had different but closely related *ftsI* alleles (Skaare et al., 2014a). Both clones are associated with increased morbidity (Skaare et al., 2014a), with ST14/IIA representing 25% of invasive rPBP3 NTHi infections in Sweden 2010–2012 (Mansson et al., 2017). The ST14/IIA clone also caused an outbreak with severe outcomes in a nursing home in Sweden (Andersson et al., 2015).

In our study, 21/113 phenotypically tested isolates were categorized as ampicillin resistant by gradient MIC. Of these, we detected *bla*_TEM–1_ in 14 and rPBP3 in six isolates, whereas one isolate had both mechanisms. Notably, one isolate expressing beta-lactamase phenotypically had a *bla*_TEM–1_ not detected by Abricate, because the assembled open reading frame (ORF) was split in two contigs. Consequently, annotation-based analysis was necessary for detection, illustrating the importance of comparing phenotypic and genotypic characterization to avoid falsely negative results.

The presence or absence of genetic resistance markers was generally associated with high positive or negative predictive values, respectively, for phenotypic susceptibility categorization as R or S/I to the tested antibiotics. A notable exception was that a significant proportion of rPBP3 isolates were categorized as susceptible to ampicillin based on MIC and clinical breakpoints. Furthermore, one ST368 isolate was positive by rPBP3 screening (PG1 zone < 6 mm and beta-lactamase negative) and phenotypically resistant to cefuroxime (gradient MIC = 4 mg/L) but lacked rPBP3-defining substitutions. The isolate had a substitution pattern (V547I, A554T, N569S, and E603N) previously observed in three respiratory ST368 isolates from Norway with similar resistance phenotype (Skaare, 2016). Interestingly, the A554T substitution is infrequent in susceptible isolates but is present in the reference strain *H. influenzae* ATCC 49247, which expresses slightly higher beta-lactam resistance levels than expected from genotyping (N526K, low-rPBP3 group II) (Skaare et al., 2010, 2014a). Transformational studies are required to clarify whether A554T confers increased resistance to beta-lactams.

There were several limitations in our study. Firstly, our dataset only contained whole-genome sequenced isolates for 245 the 407 cases reported to MSIS. Our laboratory data were therefore less complete than the MSIS data in 2017, which must be taken into consideration when interpreting the data. The cause of the discrepancy between the number of cases reported to MSIS and the number of whole-genome sequenced isolates is unknown. Underreporting and difficulties at the primary laboratories in obtaining viable isolates from the patients due to antibiotic treatment prior to sampling may be possible explanations. Secondly, since AST was only performed on 113 isolates, we lacked phenotypic resistance data for 2018–2019. In addition, MIC was routinely performed by the gradient method, and not by reference methodology (broth microdilution, BMD). Care should therefore be taken when interpreting the correlation between phenotypic and genotypic resistance. For example, ampicillin MIC is underestimated in the range close to the clinical breakpoint (Skaare et al., 2015). Consequently, the PPV of rPBP3 for prediction of ampicillin resistance in *bla*_TEM–1_ negative isolates would therefore likely be higher than observed in our study (54.4%) if AST had been performed by BMD. Thirdly, we used aggregated data from MSIS, and therefore could not link these data to the data from Labware. We had no access to data on travel history, clinical symptoms, severity, vaccination status, treatment, risk factors or place of residence. We recommend including such information in future studies.

To summarize, the invasive Hi population in Norway consisted of two major phylogenetic groups, with subclustering by serotype and ST. We identified 13 clusters of closely related isolates, of which four consisted of encapsulated isolates, but found no evidence of local outbreaks. Interestingly, although most clusters belonged to common STs among invasive Hi in other European countries and had common MLST-*ftsI* profiles, distinct “Norwegian” clusters were recognizable by worldwide core genome phylogeny. We found that acquired resistance mechanisms to beta-lactams was present in almost a quarter of all invasive Hi in Norway, and identified three previously described virulent international NTHi clones with beta-lactam resistance mechanisms (*bla*_TEM–1_-carrying ST103, rPBP3 ST14/IIA and rPBP3 ST367/IIA); these should be further studied and monitored. We recommend routine WGS-based surveillance of molecular epidemiology and antibiotic resistance as a supplement to phenotypic characterization and AST in invasive Hi.

## Data availability statement

The datasets presented in this study can be found in online repositories at the European nucleotide archive (ENA) under project accession PRJEB53447. The names of the repository/repositories and accession number(s) can be found in the article/Supplementary material.

## Ethics statement

The studies involving human participants were reviewed and approved by Regional Committees for Medical and Health Research Ethics (REK), Norway, (reference number 234529). Written informed consent from the participants’ legal guardian/next of kin was not required to participate in this study in accordance with the national legislation and the institutional requirements.

## Author contributions

AW conceived the study. AW, RT, DS, IG, and ND planned the study. ND, IG, AW, and RT collected and managed the data. RT, IG, DS, AW, and JL analyzed the data. RT drafted the manuscript. DS, IG, AW, JL, ND, and RT edited the manuscript. All authors contributed to the article and approved the submitted version.

## Footnotes

1
https://microreact.org/project/i2HEcqh6MvGiuykTahgJQF-invasive-haemophilus-influenzae-norway-2017-2021

2
https://microreact.org/project/xmN8hPaKrotfLwoZMC9PwM-invasive-haemophilus-influenzae-norway-2017-2021versus-worldwide

3
https://pubmlst.org/organisms/haemophilus-influenzae

4
https://pubmlst.org/projects/iris
