# Supplementary material for: Molecular epidemiology and antibiotic resistance profiles of invasive Haemophilus influenzae from Norway 2017–2021
Source: Front Microbiol. 2022 Aug 29;13:973257. doi: 10.3389/fmicb.2022.973257 (PMC9467436; doi:10.3389/fmicb.2022.973257)

Supplementary Material

**Table S1.** Number (per 100 000) of Invasive *Haemophilus influenzae* disease cases per age group reported to MSIS in Norway 2017-2021 (N=407).

| Age group (years) | 2017 | 2018 | 2019 | 2020 | 2021 | Mean 2017-2021 |
| --- | --- | --- | --- | --- | --- | --- |
| <1 | 2 (3.4) | 1 (1.8) | 4 (7.2) | 0 (0.0) | 2 (3.8) | 1.8 (3.2) |
| 1-9 | 1 (0.2) | 6 (1.1) | 5 (0.9) | 5 (0.9) | 9 (1.7) | 5.2 (0.9) |
| 10-19 | 6 (0.9) | 2 (0.3) | 1 (0.2) | 0 (0.0) | 1 (0.2) | 2.5 (0.3) |
| 20-29 | 4 (0.6) | 5 (0.7) | 0 (0.0) | 2 (0.3) | 4 (0.6) | 3.8 (0.4) |
| 30-39 | 6 (0.9) | 5 (0.7) | 6 (0.8) | 3 (0.4) | 2 (0.3) | 4.4 (0.6) |
| 40-49 | 9 (1.2) | 6 (0.8) | 9 (1.2) | 2 (0.3) | 4 (0.6) | 6.0 (0.8) |
| 50-59 | 8 (1.2) | 8 (1.2) | 10 (1.4) | 2 (0.3) | 7 (1.0) | 7.0 (1.0) |
| 60-69 | 25 (4.4) | 21 (3.7) | 19 (3.3) | 9 (1.5) | 10 (1.7) | 16.8 (2.9) |
| 70-79 | 25 (6.6) | 14 (3.5) | 22 (5.2) | 10 (2.3) | 6 (1.3) | 15.4 (3.7) |
| 80-89 | 25 (14.2) | 18 (10.1) | 16 (8.8) | 4 (2.2) | 8 (4.2) | 14.2 (7.9) |
| ≥90 | 9 (20.2) | 5 (11.2) | 6 (13.3) | 3 (6.6) | 5 (10.8) | 5.6 (12.4) |
| Total | 120 (2.3) | 91 (1.7) | 98 (1.8) | 40 (0.7) | 58 (1.1) | 81.4 (1.5) |

**Table S2**. Number (per 100 000) of invasive *Haemophilus influenzae* disease cases per sex reported to MSIS in Norway 2017-2021 (N=407).

| Year | Male | Female | M/F-ratio (:1) | Total |
| --- | --- | --- | --- | --- |
| 2017 | 59 (2.2) | 61 (2.3) | 1.0 | 120 (2.3) |
| 2018 | 40 (1.5) | 51 (1.9) | 0.8 | 91 (1.7) |
| 2019 | 38 (1.4) | 60 (2.3) | 0.6 | 98 (1.8) |
| 2020 | 14 (0.5) | 26 (1.0) | 0.5 | 40 (0.7) |
| 2021 | 31 (1.1) | 27 (1.0) | 1.1 | 60 (1.1) |
| Mean 2017-2021 | 36.4 (1.4) | 45.0 (1.7) | 0.8 | 81.4 (1.5) |

**Table S3.** Number of cases (per 100 000 population) of invasive *Haemophilus influenzae* disease per county and year reported to MSIS, 2017-2021 (N=406, including 1 with unknown country in 2021, one in 2019 with missing data).

| County | 2017 | 2018 | 2019 | 2020 | 2021 | Mean 2017-2021 |
| --- | --- | --- | --- | --- | --- | --- |
| Agder | 12 (4.0) | 5 (1.6) | 8 (2.6) | 3 (1.0) | 4 (1.3) | 6.4 (2.1) |
| Innlandet | 12 (3.2) | 8 (2.2) | 17 (4.6) | 3 (0.8) | 10 (2.7) | 10.0 (2.7) |
| Møre og Romsdal | 4 (1.5) | 3 (1.1) | 4 (1.5) | 2 (0.8) | 2 (0.8) | 3.0 (1.1) |
| Nordland | 3 (1.2) | 8 (3.3) | 2 (0.8) | 1 (0.4) | 1 (0.4) | 3.0 (1.2) |
| Oslo | 15 (2.2) | 10 (1.5) | 7 (1.0) | 5 (0.7) | 10 (1.4) | 9.4 (1.4) |
| Rogaland | 9 (1.9) | 8 (1.7) | 9 (1.9) | 3 (0.6) | 8 (1.7) | 7.4 (1.6) |
| Troms og Finnmark | 4 (1.6) | 3 (1.2) | 3 (1.2) | 2 (0.8) | 1 (0.4) | 2.6 (1.1) |
| Trøndelag | 14 (3.1) | 6 (1.3) | 6 (1.3) | 1 (0.2) | 3 (0.6) | 6.0 (1.3) |
| Vestfold og Telemark | 8 (1.9) | 8 (1.9) | 8 (1.9) | 2 (0.5) | 4 (0.9) | 6.0 (1.4) |
| Vestland | 14 (2.2) | 13 (2.1) | 8 (1.3) | 6 (0.9) | 5 (0.8) | 9.2 (1.5) |
| Viken | 25 (2.1) | 19 (1.6) | 25 (2.0) | 12 (1.0) | 9 (0.7) | 18.0 (1.5) |
| Total | 120 (2.3) | 91 (1.7) | 97 (1.8) | 40 (0.7) | 58 (1.1) | 81.2 (1.5) |

**Table S4.** Number (per 100 000 population) whole genome sequenced invasive *Haemophilus influenzae* isolates in Norway 2017-2021 (N=245) per sex and year.

| Year | Male | Female | M/F-ratio (:1) | Total |
| --- | --- | --- | --- | --- |
| 2017 | 6 (0.2) | 7 (0.3) | 0.9 | 13 (0.2) |
| 2018 | 21 (0.8) | 31 (1.2) | 0.7 | 52 (1.0) |
| 2019 | 35 (1.3) | 58 (2.2) | 0.6 | 93 (1.7) |
| 2020 | 13 (0.5) | 25 (0.9) | 0.5 | 38 (0.7) |
| 2021 | 26 (1.0) | 23 (0.9) | 1.1 | 49 (0.9) |
| Mean 2017-2021 | 20.2 (0.8) | 28.8 (1.1) | 0.7 | 48 (0.9) |

**Table S5.** Number (per 100 000 population) whole genome sequenced *Haemophilus influenzae* isolates (N=245) in Norway 2017-2021 per county and year.

| County | 2017 | 2018 | 2019 | 2020 | 2021 | Mean 2017-2021 |
| --- | --- | --- | --- | --- | --- | --- |
| Agder | 0 (0.0) | 4 (1.3) | 8 (2.6) | 3 (1.0) | 4 (1.3) | 4.8 (1.6) |
| Innlandet | 3 (0.8) | 4 (1.1) | 17 (4.6) | 3 (0.8) | 9 (2.4) | 7.2 (1.9) |
| Møre og Romsdal | 0 (0.0) | 3 (1.1) | 4 (1.5) | 2 (0.8) | 2 (0.8) | 2.8 (1.0) |
| Nordland | 2 (0.8) | 6 (2.5) | 1 (0.4) | 1 (0.4) | 1 (0.4) | 2.2 (0.9) |
| Oslo | 2 (0.3) | 4 (0.6) | 7 (1.0) | 5 (0.7) | 10 (1.4) | 5.6 (0.8) |
| Rogaland | 0 (0.0) | 5 (1.1) | 8 (1.7) | 2 (0.4) | 6 (1.2) | 5.3 (1.1) |
| Troms og Finnmark | 0 (0.0) | 2 (0.8) | 2 (0.8) | 2 (0.8) | 1 (0.4) | 1.8 (0.7) |
| Trøndelag | 2 (0.4) | 3 (0.6) | 6 (1.3) | 1 (0).2 | 3 (0.6) | 3.0 (0.6) |
| Vestfold og Telemark | 1 (0.2) | 3 (0.7) | 8 (1.9) | 1 (0.2) | 2 (0.5) | 3.0 (0.7) |
| Vestland | 1 (0.2) | 8 (1.3) | 6 (0.9) | 6 (0.9) | 4 (0.6) | 5.0 (0.8) |
| Viken | 0.2 (2) | 10 (0.8) | 26 (2.1) | 12 (1.0) | 7 (0.6) | 11.4 (0.9) |
| Total | 13 (0.2) | 52 (1.0) | 93 (1.7) | 38 (0.7) | 49 (0.9) | 49.0 (0.9) |

**Table S6.** Serotype distribution (number and proportion) of whole genome sequenced *Haemophilus influenzae* isolates from Norway 2017-2021 (N=245), as predicted by HiCap.

| Type | 2017  n (%) | 2018  n (%) | 2019  n (%) | 2020  n (%) | 2021  n (%) | Total  n (%) |
| --- | --- | --- | --- | --- | --- | --- |
| a | 1 (7.7) | 1 (1.9) | 3 (3.2) | 3 (7.9) | 7 (14.3) | 15 (6.1) |
| b | 0 (0.0) | 6 (11.5) | 8 (8.6) | 1 (2.6) | 3 (6.1) | 18 (7.3) |
| c | 0 (0.0) | 0 (0.0) | 0 (0.0) | 0 (0.0) | 0 (0.0) | 0 (0.0) |
| d | 0 (0.0) | 0 (0.0) | 0 (0.0) | 0 (0.0) | 0 (0.0) | 0 (0.0) |
| e | 0 (0.0) | 0 (0.0) | 3 (3.2) | 3 (7.9) | 0 (0.0) | 6 (2.4) |
| f | 2 (15.4) | 9 (17.3) | 10 (10.8) | 2 (5.3) | 7 (14.2) | 30 (12.2) |
| NT | 10 (76.9) | 36 (69.2) | 69 (74.2) | 29 (76.3) | 32 (65.3) | 176 (71.8) |
| Total | 13 (100) | 52 (100) | 93 (100) | 38 (100) | 49 (100) | 245 (100) |

**Figure S1.** Number of invasive *Haemophilus influenzae* per 100 000 population reported to MSIS in Norway 2017-2021, per sex and age group (N=407)


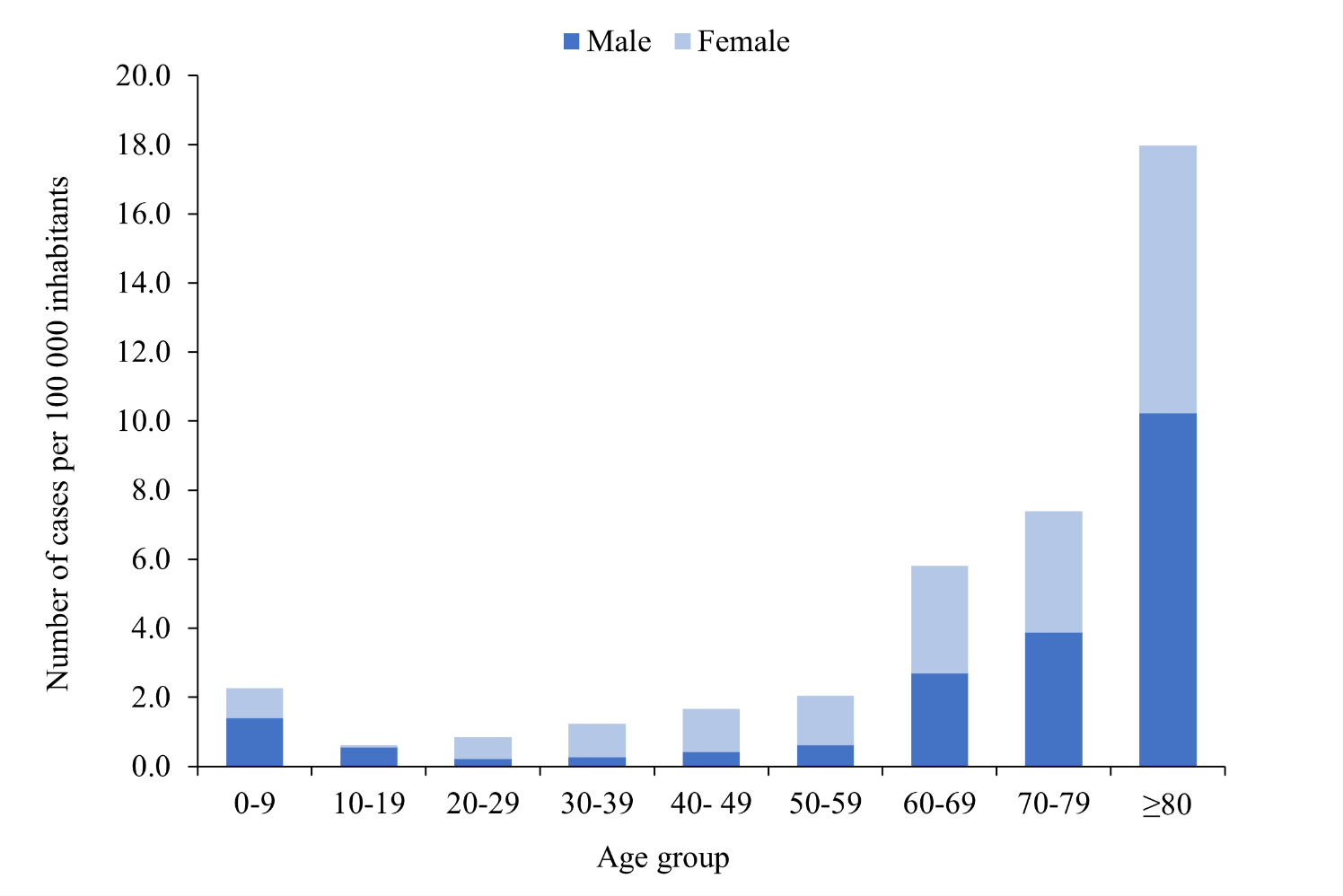


**Figure S2.** Number of invasive *Haemophilus influenzae* disease per 100 000 population reported to MSIS 2017-2021 in Norway, per county (N=406, one with missing data).


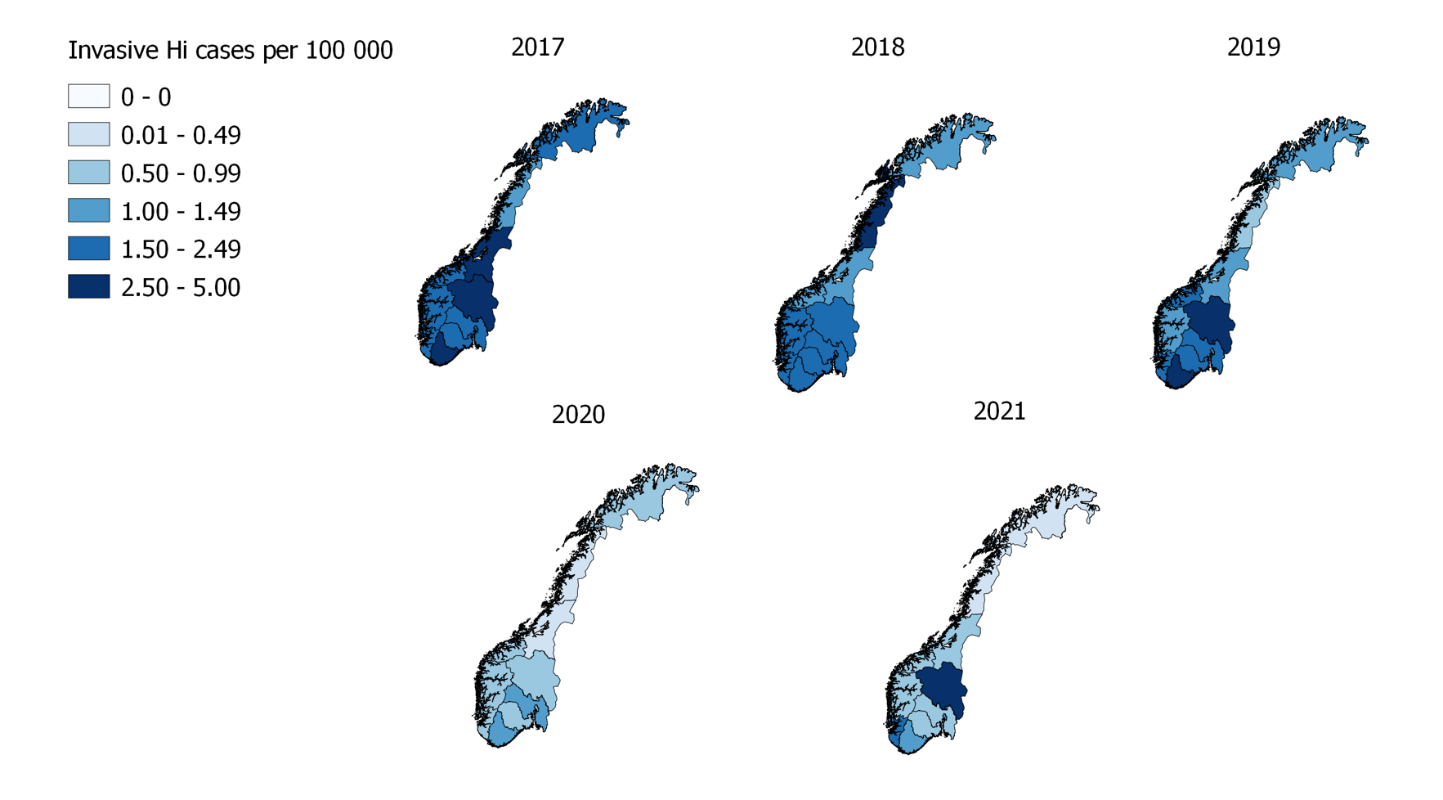

Supplement: Supplementary file 2 [file Data_Sheet_1.docx]
